# Low-pressure hydrocephalus with supratentorial and infratentorial hygromas after suboccipital decompression for cerebellar infarction

**DOI:** 10.1093/omcr/omag198

**Published:** 2026-10-25

**Authors:** Tobias A Wagner-Altendorf, Harald Krenzlin, Hannes Schacht, Georg Royl, Nils Werring, Peter Schramm, Naureen Keric, Jens Minnerup

**Affiliations:** University of Lübeck, Department of Neurology, Ratzeburger Allee 160, 23538 Lübeck, Germany; University of Lübeck, Department of Neurosurgery, Ratzeburger Allee 160, 23538 Lübeck, Germany; University of Lübeck, Department of Neuroradiology, Ratzeburger Allee 160, 23538 Ratzeburger Allee 160, 23538 Lübeck, Germany; University of Lübeck, Department of Neurology, Ratzeburger Allee 160, 23538 Lübeck, Germany; University of Lübeck, Department of Neurology, Ratzeburger Allee 160, 23538 Lübeck, Germany; University of Lübeck, Department of Neuroradiology, Ratzeburger Allee 160, 23538 Ratzeburger Allee 160, 23538 Lübeck, Germany; University of Lübeck, Department of Neurosurgery, Ratzeburger Allee 160, 23538 Lübeck, Germany; University of Lübeck, Department of Neurology, Ratzeburger Allee 160, 23538 Lübeck, Germany

A 57-year-old man presented with right-sided homonymous hemianopia, hemisensory loss, and mild right facial weakness due to left posterior cerebral artery occlusion treated with intravenous thrombolysis and mechanical thrombectomy. He subsequently developed progressive hemiparesis and anarthria caused by basilar artery occlusion with cerebellar infarction requiring repeat thrombectomy and decompressive suboccipital craniectomy.

Follow-up imaging demonstrated extensive supra- and infratentorial hygromas, with the left frontal hygroma requiring burr-hole drainage (Fig. 1A). Despite ventriculoperitoneal shunting, progressive ventricular enlargement with transependymal edema occurred (Fig. 1B). Clinically, the patient developed a severe brainstem syndrome characterized by anarthria, dysphagia, and a right-dominant tetraparesis. In addition, vertical and horizontal gaze palsy was present. The patient was intermittently somnolent and at times not arousable. Ventricular size repeatedly increased when CSF drainage was reduced despite low measured intracranial pressures, consistent with low-pressure hydrocephalus. Low-pressure hydrocephalus, although its pathophysiology is yet not fully understood, is thought to involve altered permeability and viscoelasticity of the brain parenchyma, resulting in an imbalance in CSF circulation and thus pressure dissociation between ventricular and subarachnoid CSF compartments [1–4]; craniectomy and associated CSF leakage may further disturb these pressure-volume relationships [5].

**Figure 1:**
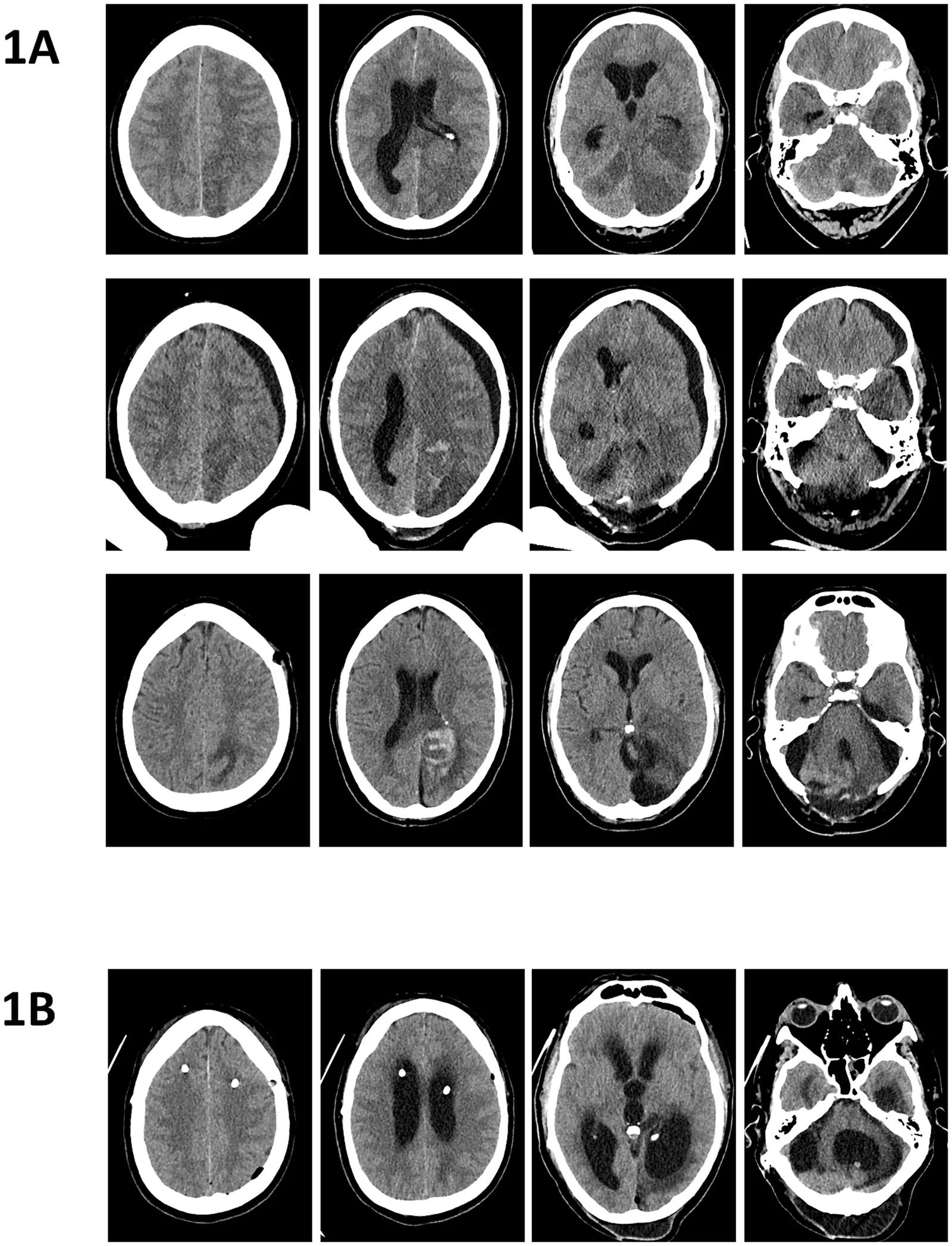
(A) Top row: Axial CT demonstrating left posterior cerebral and cerebellar infarction, before suboccipital decrompression. Middle and bottom rows: Axial CT demonstrating extensive supra- and infratentorial hygromas, before (middle row) and after (bottom row) surgical drainage of a left frontal hygroma. (B) Axial CT images obtained 6 weeks later, demonstrating marked enlargement of the lateral ventricles despite bilateral ventricular shunting, consistent with low-pressure hydrocephalus.

The condition required bilateral ventricular catheters, one connected to a programmable valve and one draining freely into the peritoneum, resulting in radiographic stabilization. As the lowest setting of the programmable valve exceeded the intracranial pressure, bilateral drainage with one catheter bypassing the valve was placed to facilitate necessary drainage due to the high drainage requirement and persistent CSF accumulation.

The level of consciousness gradually stabilized. Despite slight clinical improvement, a severe brainstem syndrome persisted. At discharge, the patient showed spontaneous eye opening without reliable visual fixation or verbal response, and persistent hemiparesis of the right side. He only rarely followed simple commands and was able to communicate using gestures with the left hand.
